# Paraneoplastic Fever in a Patient With Cervical Carcinoma: A Diagnostic Challenge and Management Approach

**DOI:** 10.1155/crom/6473606

**Published:** 2026-09-10

**Authors:** Ingrid Masilenge, Lucylight Mallya, Ravinder Jandwa, Samwel Mallango, Queen Tarimo, Kezia Tessua, Alita Mrema

**Affiliations:** ^1^ Department of Clinical Oncology, Muhimbili University of Health and Allied Sciences, Dar es Salaam, Tanzania, muchs.ac.tz; ^2^ Department of Clinical Oncology, Ocean Road Cancer Institute, Dar es salaam, Tanzania, orci.or.tz

**Keywords:** cervical cancer, naproxen challenge, neoplastic fever, paraneoplastic syndromes, Tanzania

## Abstract

**Introduction:**

Persistent fever in patients with cancer presents a significant diagnostic challenge, particularly in settings where infectious diseases are highly prevalent. Neoplastic fever is an important noninfectious cause of fever that may mimic infection and result in unnecessary investigations, antimicrobial therapy, and delays in cancer treatment.

**Case Presentation:**

We report a case of a 45‐year‐old female patient with advanced cervical carcinoma who presented with persistent high‐grade fever unresponsive to antibiotics. An extensive infectious workup was negative. Following exclusion of alternative causes, a naproxen challenge was performed, resulting in rapid resolution of fever within 24 h. Fever recurred upon withdrawal and resolved again after naproxen reintroduction, supporting the diagnosis of neoplastic fever.

**Conclusion:**

Neoplastic fever should be considered early in cancer patients with persistent unexplained fever, particularly in tuberculosis‐ and malaria‐endemic settings. Following exclusion of infection, the naproxen challenge may provide a simple and affordable diagnostic step to distinguish neoplastic fever from infectious causes, reduce unnecessary antimicrobial use, shorten diagnostic delays, and facilitate timely cancer‐directed therapy.

**Key Clinical Message**

In tuberculosis‐ and malaria‐endemic regions, neoplastic fever should be considered in cancer patients with persistent fever that remains unexplained despite appropriate evaluation and antimicrobial therapy. The naproxen challenge is a safe, inexpensive, and practical bedside tool that can support diagnosis, reduce unnecessary antimicrobial exposure, and facilitate timely oncologic management.

## 1. Introduction

Patients with cancer may develop fever from a variety of causes, including infection, neutropenia, thromboembolic disease, drug reactions, transfusion reactions, inflammatory disorders, and malignancy itself. Consequently, persistent fever in oncology patients often presents a significant diagnostic challenge and may delay definitive cancer treatment if the underlying cause is not promptly identified.

Neoplastic fever is a recognized neoplastic syndrome and one of the most common causes of fever of unknown origin in patients with cancer, accounting for approximately 15%–20% of cases [1, 2]. It is thought to result from tumor‐associated production of endogenous pyrogenic cytokines, including interleukin‐1 (IL‐1), interleukin‐6 (IL‐6), and tumor necrosis factor‐*α* (TNF‐*α*), which stimulate prostaglandin E2 synthesis and reset the hypothalamic thermoregulatory set point [2–4]. Clinically, the diagnosis is established after exclusion of infectious and other identifiable causes of fever and is supported by a characteristic rapid response to nonsteroidal anti‐inflammatory drugs (NSAIDs), particularly naproxen [5, 6].

Although neoplastic fever has been well described in hematological malignancies and several solid tumors [5, 7–10], its recognition in patients with cervical cancer may be challenging because infectious causes of fever are often considered first, especially in low‐ and middle‐income countries (LMICs) where diseases such as tuberculosis and malaria remain highly prevalent [11]. Consequently, patients may undergo extensive investigations and repeated courses of antimicrobial therapy before neoplastic fever is suspected.

This case report describes a patient with advanced cervical cancer who presented with persistent fever that remained unexplained despite comprehensive investigations and multiple empiric antimicrobial treatments. The case highlights the diagnostic challenges associated with persistent fever in cancer patients and demonstrates the value of a structured, resource‐conscious diagnostic approach, including the use of the naproxen challenge test as a simple and affordable bedside tool to support the diagnosis of neoplastic fever.

## 2. Case Presentation

A 45‐year‐old woman with a negative HIV status and no known drug allergies presented to our clinic with a 6‐month history of foul‐smelling vaginal discharge accompanied by lower abdominal pain. She denied vaginal bleeding. At presentation, her Eastern Cooperative Oncology Group (ECOG) performance status was 1. She was afebrile, mildly pale, and had no jaundice, with the remainder of the systemic examination being unremarkable.

Pelvic examination revealed a firm cervical mass with irregular margins extending to the upper two‐thirds of the vagina. A cervical biopsy was performed, and histopathological examination confirmed nonkeratinizing squamous cell carcinoma of the cervix. Magnetic resonance imaging (MRI) of the abdomen and pelvis demonstrated a heterogeneous, ill‐defined enhancing cervical mass measuring 5.17 × 6.78 × 7.10 cm with parametrial extension to the lower vagina, consistent with International Federation of Gynecology and Obstetrics (FIGO) Stage IIIA disease. Chest imaging showed no evidence of intrathoracic pathology.

Initial laboratory investigations revealed leukocytosis (14.28 × 10^9^/L) and moderate microcytic hypochromic anemia, whereas liver and renal function tests were within normal limits. In view of the foul‐smelling vaginal discharge and suspected superimposed genital tract infection, oral amoxicillin–clavulanic acid 625 mg every 12 h was prescribed for 5 days.

One week later, the patient developed recurrent high‐grade fevers occurring every 2–3 days that were unresponsive to acetaminophen. Repeat complete blood count demonstrated worsening leukocytosis (19.00 × 10^9^/L), and a malaria rapid diagnostic test (MRDT) was positive. She was treated with artemether 20 mg + lumefantrine 120 mg tablets twice daily for 3 days and injection ceftriaxone + sulbactam 1.5 g twice daily for 5 days. A repeat MRDT after completion of therapy was negative; however, the fever persisted and repeat laboratory testing continued to demonstrate leukocytosis.

**Figure 1:**
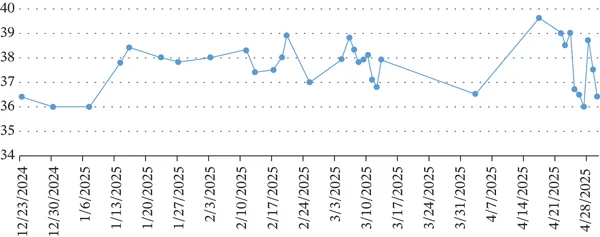
Trend of body temperature before initiation of naproxen therapy.

**Figure 2:**
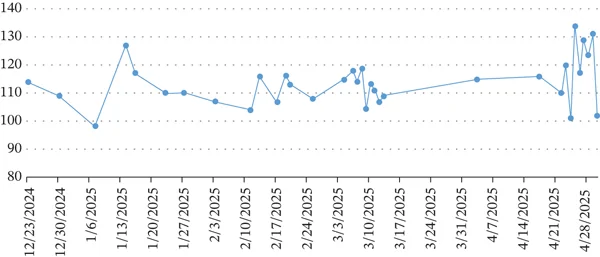
Trend of heart rate before initiation of naproxen therapy.

Over the ensuing weeks, she continued to experience recurrent fever accompanied by persistent tachycardia (Figures 1 and 2), drenching night sweats, generalized weakness, headache, and mild vaginal bleeding. The lowest recorded hemoglobin was 7.1 g/dL, whereas the lowest leukocyte count remained elevated at 15.74 × 10^9^/L. Despite sequential treatment with multiple antimicrobial agents, including amoxicillin–clavulanic acid tablets 625 mg every 12 h for 5 days, intravenous meropenem 1 g every 8 h for 5 days, and intravenous polymyxin B every 12 h for 5 days, there was no clinical improvement.

Serum C‐reactive protein (CRP) was elevated at 264 mg/L. Peripheral blood smear demonstrated normocytic normochromic anemia with features suggestive of an underlying inflammatory or infectious process. Blood culture and sensitivity, high vaginal swab (HVS), urine culture, sputum GeneXpert, and Hepatitis B surface antigen testing were all negative. Blood cultures were not consistently obtained during peak febrile episodes, and echocardiography to exclude nonbacterial thrombotic endocarditis was not performed. Table 1

**Table 1:** Table summarizing the diagnostic workflow, evaluation, and exclusion of infectious etiologies.

Date	Investigation
23/12/2024	Biopsy (cervical ca), HIV serology
30/12/2024	MRI abd&pel, CXR, FBP, RFT, LFT
14/01/2025	MRDT (positive)
23/01/2025	MRDT (negative)
13/02/2025	Blood culture and sensitivity (no growth)
10/03/2025	GeneXpert
07/03/2025	Hep B, Hep C, blood slide for malaria
11/03/2025	CRP, peripheral smear
03/04/2025	High vaginal swab, urine analysis and culture

Given the high prevalence of tuberculosis in our setting and the patient′s persistent fever and drenching night sweats, empirical antituberculosis therapy was initiated despite negative initial microbiological investigations. Three weeks after starting treatment, she continued to experience persistent high‐grade fever and constitutional symptoms without clinical improvement. Owing to the perceived lack of benefit, she discontinued the antituberculosis therapy.

**Figure 3:**
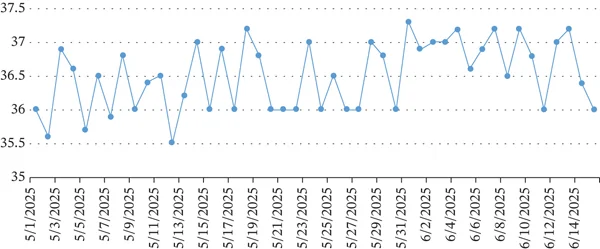
Trend of body temperature following initiation of naproxen therapy.

**Figure 4:**
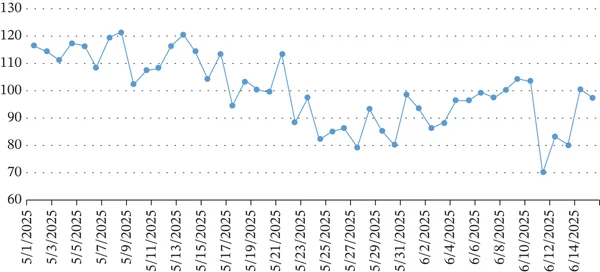
Trend of heart rate following initiation of naproxen therapy.

Oral fluconazole 150 mg was initiated once daily every 72 h for three doses, followed by once weekly for 6 weeks, because of persistent vaginal discharge suggestive of vulvovaginal candidiasis. Despite treatment, the patient continued to experience recurrent fever. Following exclusion of infectious and other alternative causes, neoplastic fever was suspected approximately 4 months after the initial diagnosis of cervical cancer. A naproxen challenge was undertaken, and naproxen 500 mg twice daily was initiated on April 24, 2025, approximately 4 months after the initial diagnosis resulting in the rapid resolution of fever and night sweats within 24 h. Fever recurred within 12 h of naproxen withdrawal and was accompanied by delirium, but both symptoms resolved within 24 h after reintroduction of naproxen, strongly supporting the diagnosis of neoplastic fever (Figure 3). In contrast, the patient′s heart rate showed a more gradual improvement, declining approximately 2 weeks after initiation of naproxen therapy (Figure 4).

**Figure 5:**
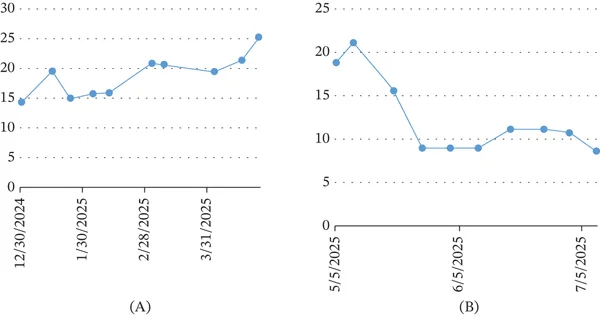
Trend of leucocyte count (A) before and (B) after initiation of naproxen therapy.

**Figure 6:**
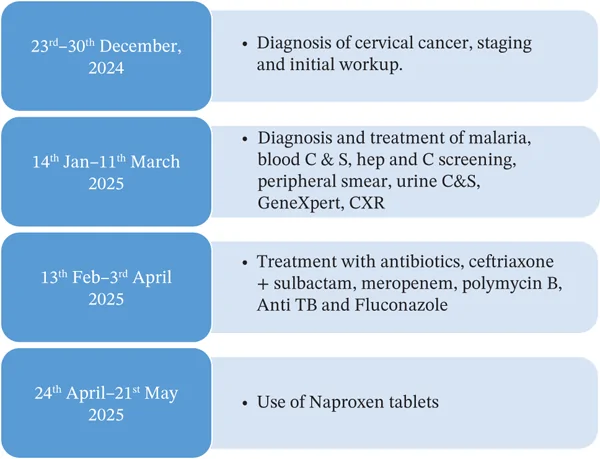
Timeline of the patient′s clinical course, diagnostic evaluation, and management.

The patient was treated with external beam radiotherapy (EBRT) to a planned dose of 50 Gy in 20 fractions, followed by three fractions of intracavitary brachytherapy at 8 Gy each. Naproxen was continued for 21 days and was discontinued during the 13th fraction of EBRT (32.5 Gy of the planned 50 Gy) without recurrence of fever. Although the patient′s fever resolved rapidly following naproxen initiation, the leucocyte count remained elevated throughout naproxen therapy and normalized only after the 16th fraction of EBRT, corresponding to approximately 40 Gy (Figure 5). A timeline of the events is presented in Figure 6.

## 3. Discussion

We report a case of neoplastic fever in a woman diagnosed with advanced cervical cancer who developed persistent high‐grade fever shortly after presentation to our facility. Over a period of 4 months, she experienced recurrent fevers exceeding 38°C without a discernible pattern. Extensive evaluation, including physical examination, laboratory investigations, microbiological cultures, and imaging, failed to identify an infectious or alternative etiology. The patient had not received prior cancer‐directed therapy, had no history of transfusion reactions, and was not taking medications commonly associated with drug‐induced fever, thereby excluding other common causes of fever in oncology patients. Despite multiple empiric antimicrobial regimens, including antimalarial, broad‐spectrum antibacterial, and antituberculosis therapies, the fever persisted.

Although neoplastic fever is a recognized paraneoplastic syndrome [1, 2], distinguishing it from infection remains a significant clinical challenge, particularly in patients with advanced cancer. This challenge is even greater in LMICs where infectious diseases such as tuberculosis, malaria, and bacterial infections are common and frequently represent the initial diagnostic consideration [11]. Consequently, patients may undergo prolonged investigations and repeated courses of antimicrobial therapy before neoplastic fever is considered.

This case has several limitations that warrant acknowledgment. Blood cultures were not consistently obtained during peak febrile episodes, and echocardiography was not performed to exclude nonbacterial thrombotic endocarditis because of resource limitations in our clinical setting. Nevertheless, the diagnosis of neoplastic fever remains well supported, as the patient met the established diagnostic criteria, including persistent fever despite appropriate antimicrobial therapy, exclusion of common infectious causes based on available investigations, and a rapid response to naproxen [5, 6]. In this context, the naproxen challenge provided an important additional diagnostic aid, helping to strengthen the diagnosis despite these diagnostic gaps. The reproducible resolution of fever following naproxen initiation, recurrence upon its withdrawal, and subsequent resolution after reintroduction further strengthened the diagnosis. This response pattern has been reported as a useful diagnostic aid in distinguishing neoplastic fever from infectious causes after appropriate exclusion of active infection [3, 6, 12–14].

Another noteworthy observation in this case was the dissociation between resolution of fever and normalization of leukocytosis. Although fever resolved rapidly following naproxen administration, leukocytosis persisted until the patient had received approximately 40 Gy of radiotherapy (Figure 5). This finding suggests that fever and leukocytosis may be mediated through overlapping but distinct biological pathways. The rapid resolution of fever is consistent with naproxen‐mediated suppression of prostaglandin‐dependent thermoregulation, whereas the persistent leukocytosis may have reflected ongoing tumor‐driven inflammatory cytokine production that improved only after a substantial reduction in tumor burden with radiotherapy. Tumor lysis syndrome and radiation‐induced inflammatory response were considered as possible contributors to delayed leukocyte normalization; however, these mechanisms could not be confirmed in the absence of specific supporting investigations.

Beyond these diagnostic considerations, the principal value of this case lies not in describing neoplastic fever itself, but in demonstrating a practical, resource‐conscious diagnostic approach. Our patient received multiple antimicrobial regimens without clinical improvement and underwent extensive investigations before neoplastic fever was suspected. This experience underscores the importance of systematically reassessing the diagnosis when persistent fever remains unexplained despite appropriate evaluation and treatment.

Another major clinical implication of this case is the continued relevance of the naproxen challenge test. Despite being well described in the literature, it remains underutilized in routine practice. In resource‐constrained settings, the naproxen challenge represents a simple, inexpensive, and widely available bedside tool that may assist clinicians in distinguishing neoplastic fever from infectious causes after appropriate exclusion of active infection.

Therefore, in settings where infectious diseases are highly prevalent, clinicians should maintain a high index of suspicion for neoplastic fever in cancer patients with persistent fever that remains unexplained after appropriate evaluation. This case highlights the value of a structured, low‐cost diagnostic approach in which the naproxen challenge serves as a safe, affordable, and readily available bedside tool. Earlier use of this strategy may shorten diagnostic delays, avoid extensive investigations, reduce unnecessary antimicrobial exposure, and facilitate timely cancer‐directed treatment.

## 4. Conclusion

Neoplastic fever should be considered early in cancer patients with persistent fever that remains unexplained despite appropriate investigation and antimicrobial therapy. In settings where infectious diseases such as tuberculosis and malaria are highly prevalent, delayed recognition may lead to unnecessary treatments and prolonged diagnostic workups. When infection has been appropriately excluded, a naproxen challenge should be considered as a simple, affordable, and clinically useful step in evaluating suspected neoplastic fever. It may help distinguish neoplastic fever from infectious causes, facilitating earlier diagnosis, reducing unnecessary antimicrobial use, and enabling timely cancer‐directed treatment.

## Author Contributions

Conception: Ingrid Masilenge and Lucylight Mallya. Acquisition of clinical data: Ravinder Jandwa and Samwel Mallango. Drafting and revision of work: Alita Mrema, Kezia Tessua, and Queen Tarimo.

## Funding

No funding was received for this manuscript.

## Disclosure

All authors have read and approved the final version of the manuscript.

## Ethics Statement

This case report was exempted from clinical approval from our institution, as this paper reports a single case that emerged during normal clinical practice.

## Consent

Written informed consent was obtained from the patient for the publication of this case report and accompanying images.

## Conflicts of Interest

The authors declare no conflicts of interest.

## Data Availability

The data that support the findings of this study are available on request from the corresponding author. The data are not publicly available due to privacy or ethical restrictions.
